# Supplementary material for: Methods Combining Genomic and Epidemiological Data in the Reconstruction of Transmission Trees: A Systematic Review
Source: Pathogens. 2022 Feb 15;11(2):252. doi: 10.3390/pathogens11020252 (PMC8875843; doi:10.3390/pathogens11020252)
Supplement: Supplementary file 1 [file pathogens-11-00252-s001.zip › pathogens-1524525-supplementary.pdf]

Table S1. Excluded articles and reasons for exclusion.

	Title	Reference	Reasons for Exclusion
1	Source identification in two criminal cases using phylogenetic analysis of HIV-1 DNA sequences	Scaduto et al., 2010 [69]	Only uses genetic data to infer transmission
2	High-resolution typing by integration of genome sequencing data in a large tuberculosis cluster	Schurch et al., 2010 [70]	No formal combination of genetic and epidemiological data
3	Phylodynamic analysis of a viral infection network	Schiino, 2012 [71]	Review on transmission clusters
4	Combining epidemiological and genetic networks signifies the importance of early treatment in HIV-1 transmission	Zarrabi et al., 2012 [72]	Graphical representation of contact info with a SNP cut-off
5	Detectable signals of episodic risk effects on acute HIV transmission systems using genetic data	Alam et al., 2013 [73]	Only uses a transmission model and not genetic data
6	Inferring the inter-host transmission of influenza A virus using patterns of intra-host genetic variation	Stack et al., 2013 [74]	Cannot use consensus sequence
7	Inferring the source of transmission with phylogenetic data	Volz et al., 2013 [15]	Uses simulated data
8	Bayesian Inference of Sampled Ancestor Trees for Epidemiology and Fossil Calibration	Gavryushkina et al., 2014 [75]	Reconstructs a phylogenetic tree with sampled ancestors and not a transmission tree
9	Marked microevolution of a unique Mycobacterium tuberculosis strain in 17 years of ongoing transmission in a high risk population	Mehaffy et al., 2014 [76]	No formal combination of genetic and epidemiological data
10	Two-phase importance sampling for inference about transmission trees	Numminen et al., 2014 [77]	Each data cluster was analyzed independently from others
11	Within-host bacterial diversity hinders accurate reconstruction of transmission networks from genomic distance data	Worby et al., 2014 [22]	Uses only (simulated) genetic data
12	The application of genomics to tracing bacterial pathogen transmission	Croucher et Didelot, 2015 [78]	General overview of use of WGS
13	Phylogenetic visualization of the spread of H7 influenza A viruses	Janies et al., 2015 [79]	Phylogeographic reconstruction represented by a transmission network
14	The impact of within-herd genetic variation upon inferred transmission trees for foot-and-mouth disease virus	Valdazo-Gonzalez et al., 2015 [80]	Only uses genetic data to infer transmission
15	Ebola Virus Epidemiology and Evolution in Nigeria	Folarin et al., 2016 [81]	No formal combination of genetic and epidemiological data
16	Using genomics data to reconstruct transmission trees during disease outbreaks	Hall et al., 2016 [82]	Review
17	Tracing Origins of the Salmonella Bareilly Strain Causing a Food-borne Outbreak in the United States	Hoffman et al., 2016 [83]	Geographic mapping of a phylogeny
18	Molecular Infectious Disease Epidemiology: Survival Analysis and Algorithms Linking Phylogenies to Transmission Trees	Kenah et al., 2016 [84]	Enumerates transmission trees compatible with phylogeny and epidemiological data, doesn't select
19	Infectious Disease Dynamics in Heterogeneous Landscapes	Parratt et al., 2016 [85]	Review
20	Network inference from multimodal data: A review of approaches from infectious disease transmission	Ray et al., 2016 [86]	Review
21	Ecological dynamics of influenza A viruses: cross-species transmission and global migration	Ren et al., 2016 [87]	Phylogeographic reconstruction represented by a transmission network
22	Genomic Analysis of Viral Outbreaks	Wohl et al., 2016 [88]	Review
23	Epidemiological and Evolutionary Inference of the Transmission Network of the 2014 Highly Pathogenic Avian Influenza H5N2 Outbreak in British Columbia, Canada	Xu et al., 2016 [89]	Compares two network one from genetic data and the other from epidemiological data

24	Transmission patterns and evolution of respiratory syncytial virus in a community outbreak identified by genomic analysis	Agoti et al., 2017 [90]	Only uses genetic data to infer transmission
25	Emerging Concepts of Data Integration in Pathogen Phylodynamics	Baele et al., 2017 [91]	Review
26	Inference of genetic relatedness between viral quasispecies from sequencing data	Glebova et al., 2017 [92]	Only uses genetic data to infer transmission and cannot use consensus sequence
27	Integrated genomic and interfacility patient-transfer data reveal the transmission pathways of multidrug-resistant Klebsiella pneumoniae in a regional outbreak	Snitkin et al., 2017 [93]	Phylogeographic reconstruction represented by a transmission network
28	Shared Genomic Variants: Identification of Transmission Routes Using Pathogen Deep-Sequence Data	Worby et al., 2017 [94]	Only uses deep sequencing data data to infer transmission
29	outbreaker2: a modular platform for outbreak reconstruction	Campbell et al., 2018 [95]	Implements Transphylo method into outbreaker2 using simulated data (phybreak package)
30	Genomic epidemiology for microbial evolutionary studies and the use of Oxford Nanopore sequencing technology	Choi et al., 2018 [96]	Review
31	Bayesian reconstruction of transmission within outbreaks using genomic variants	De Maio et al., 2018 [61]	Only at a single locus resolution (deep sequencing data), cannot use consensus sequence
32	Tracking a serial killer: Integrating phylogenetic relationships, epidemiology, and geography for two invasive meningococcal disease outbreaks	Ezeoke et al., 2018 [97]	Phylogeographic reconstruction represented by a transmission network
33	Multi-step genomic dissection of a suspected intra-hospital Helicobacter cinaedi outbreak	Gotoh et al., 2018 [98]	No formal combination of genetic and epidemiological data
34	Estimating Transmission from Genetic and Epidemiological Data: A Metric to Compare Transmission Trees	Kendall et al., 2018 [99]	Transmission tree comparaison
35	Phylogenetic patterns recover known HIV epidemiological relationships and reveal common transmission of multiple variants	Leitner et Romero-Severson, 2018 [100]	Uses topology of phylogenetic trees to determine whether direct transmission occured
36	The relationship between transmission time and clustering methods in Mycobacterium tuberculosis epidemiology	Meehan et al., 2018 [101]	Estimates transmission events with the age difference between MRCA node and youngest node in a cluster
37	When are pathogen genome sequences informative of transmission events?	Campbell et al., 2018 [20]	Uses simulated data to compare two methods
38	Multihospital Outbreak of a Middle East Respiratory Syndrome Coronavirus Deletion Variant, Jordan: A Molecular, Serologic, and Epidemiologic Investigation	Payne et al., 2018 [102]	Only uses epidemiological data to infer transmission
39	Nosocomial transmission of influenza: A retrospective cross-sectional study using next generation sequencing at a hospital in England (2012-2014)	Blackburn et al., 2019 [103]	No formal combination of genetic and epidemiological data
40	Inferring epidemiological links from deep sequencing data: a statistical learning approach for human, animal and plant diseases	Alamil et al., 2019 [62]	Deep sequencing data
41	Introduction of Ebola virus into a remote border district of Sierra Leone, 2014: use of field epidemiology and RNA sequencing to describe chains of transmission	DeSilva et al., 2019 [104]	Only uses epidemiological data to infer transmission
42	Reconstructing foot-and-mouth disease outbreaks: a methods comparison of transmission network models	Firestone et al., 2019 [105]	Transmission tree comparaison on simulated data

43	Whole genome sequencing for improved understanding of Mycobacterium tuberculosis transmission in a remote circumpolar region	Guthrie et al., 2019 [106]	No formal combination of genetic and epidemiological data
44	Transmission Trees on a Known Pathogen Phylogeny: Enumeration and Sampling	Hall et Colijn, 2019 [107]	Aim is not to reconstruct a transmission tree
45	Improved characterisation of MRSA transmission using within-host bacterial sequence diversity	Hall et al., 2019 [108]	Only uses genetic data to infer transmission
46	Inferring HIV-1 transmission networks and sources of epidemic spread in Africa with deep-sequence phylogenetic analysis	Ratmann et al., 2019 [109]	Deep sequencing data
47	SharpTNI: Counting and Sampling Parsimonious Transmission Networks under a Weak Bottleneck	Sashittal et al., 2019 [110]	Similar to Hall et Colijn, 2019
48	Tracing local and regional clusters of carbapenemase-producing Klebsiella pneumoniae ST512 with whole genome sequencing, Finland, 2013 to 2018	van Beek et al., 2019 [111]	No formal combination of genetic and epidemiological data + uses allele differences
49	Estimating Epidemic Incidence and Prevalence from Genomic Data	Vaughan et al., 2019 [112]	No difference between phylogeny and transmission tree
50	Phylogenetic and Demographic Characterization of Directed HIV-1 Transmission Using Deep Sequences from High-Risk and General Population Cohorts/Groups in Uganda	Bbosa et al., 2020 [113]	Deep sequencing data
51	StrainHub: a phylogenetic tool to construct pathogen transmission networks	de Bernardi Schneider et al., 2020 [114]	Phylogeographic reconstruction represented by a transmission network
52	TNet: Phylogeny-Based Inference of Disease Transmission Networks Using Within-Host Strain Diversity	Dhar et al., 2020 [115]	Deep sequencing data
53	Modeling Missing Cases and Transmission Links in Networks of Extensively Drug-Resistant Tuberculosis in KwaZulu-Natal, South Africa	Nelson et al., 2020 [116] a	Compares an empirical network (> or = to 5 SNPs difference) with network reconstructed using ergm with different missing cases scenarios
54	Social Mixing and Clinical Features Linked With Transmission in a Network of Extensively Drug-resistant Tuberculosis Cases in KwaZulu-Natal, South Africa	Nelson et al., 2020 [117] b	Graphical representation of contact info with a SNP cut-off
55	Nosocomial outbreak of COVID-19 pneumonia in Wuhan, China	Wang et al., 2020 [118]	Only uses epidemiological data to infer transmission
56	The emergence of SARS-CoV-2 in Europe and North America	Worobey et al., 2020 [119]	Compares simulated data to obtained sequence data then took a phylogeographic approach

Table S2. Pathogen studied in the selected articles and their estimated mutation rate.

Pathogen	Mutation Rate
FMDV	2.1×10^{-5}/site/day [2]
H7N7	HA and NA gene ~1×10^{-2}<br>AND PB2 gene 0.54×10^{-2}/site/year [120]
H1N1	HA and NA gene<br>$3.6 \times 10^{-3/}$site/year [121]
H5N8	6.68×10^{-3}/site/year [122]
H3N8	HA gene<br>1.74×10^{-3}/site/year [123]
RABV	2.9×10^{-4}/site/year [124]
SARS-CoV1	5.7×10^{-6}/site/day [125]
SARS-CoV2	9.90×10^{-4}/site/year [126]
HIV	*env* gene 7.4×10^{-3}/site/year [127]
Ebola virus	~2×10^{-3}/site/year [128]
poliovirus	1.1×10^{-2} /site/year [129]
BVDV	3.9×10^{-3} /site/year [33]
Salmon isavirus	HE gene<br>[$6.1 x 10^{-6}$; $1.13 x 10^{-3}$]/site/year [130,131]
Myxovirus parotidis	2.24×10^{-3}/site/year [54]
Mycobacterium tuberculosis	1.15×10^{-7}/site/year [17]
Acinetobacter baumanii	10^{-6} mutation per cell division [132]
Klebsiella pneumoniae	3.65×10^{-6}/site/year [34]
Staphylococcus areus	3×10^{-6}/site/year [133]

Table S3. Pathogen sequences studied by methods in the non-phylogenetic family. First, we recorded the study time period (D stands for days, M for months and Y for years) then the number of sequences and finally the size of the genome studied either in sequence length in nucleotides (N) or the number of single nucleotide polymorphisms (SNP). CT stands for computational time and NF, for information not found.

Methods	Pathogen	Time-period	Number of Sequences	Genome Size	References	CT	Epidemiological Unit
Aldrin 2011	*Salmon isavirus*	6 Y	61	891 N	[28]	NF	Farm
Seqtrack	H1N1	105 D	433	3,025 N	[16]	NF	Individual
	H3N8	56 D	48	903 N	[45]		Individual
	Mycobacterium tuberculosis	15 Y	1687	~4.1 x 10^6 N	[46]		Individual
	Klebsiella pneumoniae	3 Y	71	~5.5 x 10^6 N	[47]		Individual
Ypma 2012	H7N7	51 D	185	5,354 N	[32]	NF	Farm
Outbreaker	SARS-CoV1	48 D	13	29,731 N	[24]	NF	Individual
	Acinetobacter baumanii	9 M	19	1207 SNP	[36]		Individual
		3 Y	42	4,461,520 N	[35]		Individual
	Klebsiella pneumoniae	305 D	34	51 SNP	[34]		Individual
	BVDV	~2 Y	12	381 N	[33]		
Worby 2014	*MRSA*	300 D	35	~2.8 x 10^6 N	[37]	NF	Individual
Famulare 2015	Ebola	25 D	78	~19,000 N	[38]	NF	Individual
	H1N1	105 D	433	3025 N			
	poliovirus	~6 Y	358	~2600 N			
Bitrugs	*MRSA*	300 D	35	~2.8 x 10^6 N	[6]	NF	Individual
		~3 Y	40	345 SNP	[60]		
			16	209 SNP	[60]		
Outbreaker2	SARS-CoV1	48 D	13	29,731 N	[30]		Individual
	Simulated data		20		[95]	4.5 s/1000 iterations	/

Table S4. Pathogen sequences studied by methods in the sequential phylogenetic family. First, we recorded the study time period (D stands for days, M for months and Y for years) then the number of sequences and finally the size of the genome studied either in sequence length in nucleotides (N) or the number of single nucleotide polymorphisms (SNP). CT stands for computational time and NF, for information not found.

Methods	Pathogen	Time-period	Number of Sequences	Genome Size	References	CT	Epidemiological Unit
Cottam 2008	FMDV	96 D	22	8,176 N	[2]	NF	Farm
Didelot 2014	*Mycobacterium tuberculosis*	~3 Y	33	20 SNP	[17]	100,000 iterations in 5 min	Individual
Eldoholm 2016	*Mycobacterium tuberculosis*	13 Y	252	509 SNP	[39]	NF	Individual
Transphylo	SARS-CoV2	~60 D	208	29,718 N	[53]		Individual
	Myxovirus parotidis	~1 Y	24	51 SNP	[54]		Individual
	Mycobacterium tuberculosis	13 Y	86	85 SNP	[40]		Individual
		7 Y	21	134 SNP	[48]		
		19 Y	1857	~4.1 x 10^6 N	[50]		Individual
		3 Y	117		[49]		Individual
		~15 Y	329	269 SNP	[55]		Individual
	Klebsiella pneumoniae	~3 Y	73	~5.5 x 10^6 N	[51]		Individual
		14 M	82		[52]		
	Simulated data		20		[95]	4.6 s/1000 iterations	/
TiTUS	HIV	18 Y	212	1620 N	[31]	NF	Individual

Table S5. Pathogen sequences studied by methods in the simultaneous phylogenetic family. First, we recorded the study time period (D stands for days, M for months and Y for years) then the number of sequences and finally the size of the genome studied either in sequence length in nucleotides (N) or the number of single nucleotide polymorphisms (SNP). CT stands for computational time and NF, for information not found.

Methods	Pathogen	Time-period	Number of Sequences	Genome Size	References	CT	Epidemiological Unit
Ypma 2013	FMDV	64 D	12	8176 N	[5]	NF	Farm
Beastlier	H7N7	51 D	185	5354 N	[18]	NF	Farm
	H5N8		37	83 SNP	[56]		Farm
SCOTTI	FMDV	57 D	11	8176 N	[41]		Farm
	HIV				[134]		Individual
	Klebsiella pneumoniae	305 D	34	51 SNP	[41]		Individual
	Simulated data		100		[41]	1-2 h	/
Phybreak	FMDV	57 D	11	30 SNP	[26]		Farm
		65 D	15	58 SNP			
	H7N7	71 D	241	293 N		7 h/ 25000 iterations	Farm
	Mycobacterium tuberculosis	~3 Y	33	21 SNP		30 min/ 25000 iterations	Individual
	MRSA	204 D	36	27 SNP		30 min/ 25000 iterations	Individual
	Simulated data		50	10,000 N		1 min/1000 iterations	/
Morelli 2012	FMDV	57 D	10	8176 N	[23]	NF	Farm
		65 D	15				
Mollentze 2014	RABV	464 D	176	760 N	[1]	NF	Individual
Lau 2015	FMDV	64 D	12	8176 N	[42]		Farm
	Simulated data	~3 M	104	7667 N	[56]		
			150	8000 N	[42]	400,000 iterations 17.7 h	/
BORIS	FMDV	2.5 M	104	7667 N	[29]	NF	Farm
Montazeri 2020	HIV	~15 Y	19	~3000 N	[43]		Individual
	Ebola	~5 M	78	~19,000 N			
	Simulated data		50	3000		3 h	

Table S6. Details found in online instruction manuals.

Family	Package Name	Details on Epidemiological Data Format	Set Parameters	Estimated Parameters
Non-phylogenetic	Seqtrack	/	mu: mutation rate per site per day<br>haplo.length: sequence length in number of nucleotides<br>prox.mat: proximity matrix in case of ties (e.g. spatial distances)	/
	outbreaker	/	w_dens: generation time distribution<br>f_dens: sampling interval distribution	mu: mutation rate (Exponential)<br>pi: reporting probability (Beta)
	bitrugs (removed from the CRAN repository)	/	/	z: test sensitivity (Beta)<br>p: importation probability (Beta)<br>beta: transmission rate (Exponential)<br>gamma: within host/group genetic diversity (Beta)<br>gamma_gl: between host/group genetic diversity (Beta)<br>genpar: group clustering or transmission chain parameter (Exponential)
	outbreaker2	Non-dated non-oriented contact data	w_dens: generation time distribution<br>f_dens: sampling interval distribution	mu: mutation rate (Exponential)<br>pi: reporting probability (Beta)<br>eps: contact reporting coverage (Beta)<br>lambda: noninfectious contact rate (Beta)
Sequential phylogenetic	Transphylo	/	dateT: end of outbreak observation	off.r/off.p: offspring distribution (Negative Binomial)<br>w.shape/w.scale: generation time distribution (Gamma)<br>neg: average time of coalescence of two lineages<br>pi: probability of sampling<br>ws.shape/ws.scale: sampling distribution (Gamma)
Simultaneous phylogenetic	beastlier	/	/	k: Spatial kernel function<br>i: Infectious period distribution<br>l: Latent period distribution
	SCOTTI	/	/	maxHosts: maximum number of hosts in the outbreak
	phybreak (removed from the CRAN repository)	/	wh.model: within-host model	gen.mean (est.)/ gen.shape (fixed): generation interval distribution (Gamma)<br>sample.mean (est.)/ sample.shape (fixed): sampling interval distribution (Gamma)<br>wh.slope: slope of the within-host pathogen population growth<br>mu: mutation rate
